# Comparison of 4DryField PH vs. seprafilm in reducing incidence of adhesion and preventing ASBO following abdominopelvic surgery: a systematic review

**DOI:** 10.3389/fsurg.2026.1729548

**Published:** 2026-03-13

**Authors:** Shagufa Haji Cassim, Taha Luqman Bilgrami, Md Rezaul Karim, Bijendra Patel

**Affiliations:** Barts Cancer Institute, Queen Mary University of London, London, England

**Keywords:** 4DryField PH, abdominopelvic surgery, adhesiolysis, adhesions, adhesive small bowel obstruction, anti-adhesive barriers, ASBO, chronic pain

## Abstract

**Background:**

Adhesions form between tissue surfaces due to inflammation and trauma to tissue. Adhesions are a common postoperative complication of abdominopelvic surgery, often leading to chronic pain, adhesive small bowel obstruction, and increased healthcare costs. Anti-adhesive agents such as Seprafilm and 4DryField PH are employed to mitigate these risks, but direct comparisons between these barriers are lacking, creating a need to evaluate their relative efficacy. This systematic review aims to compare the efficacy of 4DryField PH and Seprafilm in preventing postoperative adhesion formation and associated complications such as ASBO in patients undergoing abdominopelvic surgery.

**Methods:**

A search of PubMed, Scopus, Cochrane Library, Embase, and trial registries identified 7 studies, including RCTs and cohort studies. Inclusion criteria were studies involving patients aged 18 and above who underwent abdominopelvic surgery and received either 4DryField PH or Seprafilm. Studies that included patients with extensive pre-existing abdominal conditions and malignancies were excluded. Data was extracted on adhesion incidence, severity, extent, chronic pain, and ASBO.

**Results:**

4DryField PH consistently reduced adhesion burden in second-look studies and showed no recurrent ASBO in one cohort. Seprafilm demonstrated modest benefit in one colorectal study but not in another. Due to non-overlapping populations, direct comparison was not possible. Exploratory trends favoured both agents over no barrier.

**Conclusion:**

Current evidence suggests that 4DryField PH may reduce adhesion burden in settings where second-look laparoscopy is performed, while Seprafilm may offer modest clinical benefit in selected colorectal surgery contexts. Because no head-to-head trials exist and the study populations differ, these findings remain hypothesis-generating. Further multicentre trials with standardised outcomes are needed to define their comparative roles.

## Introduction

**Figure 1:**
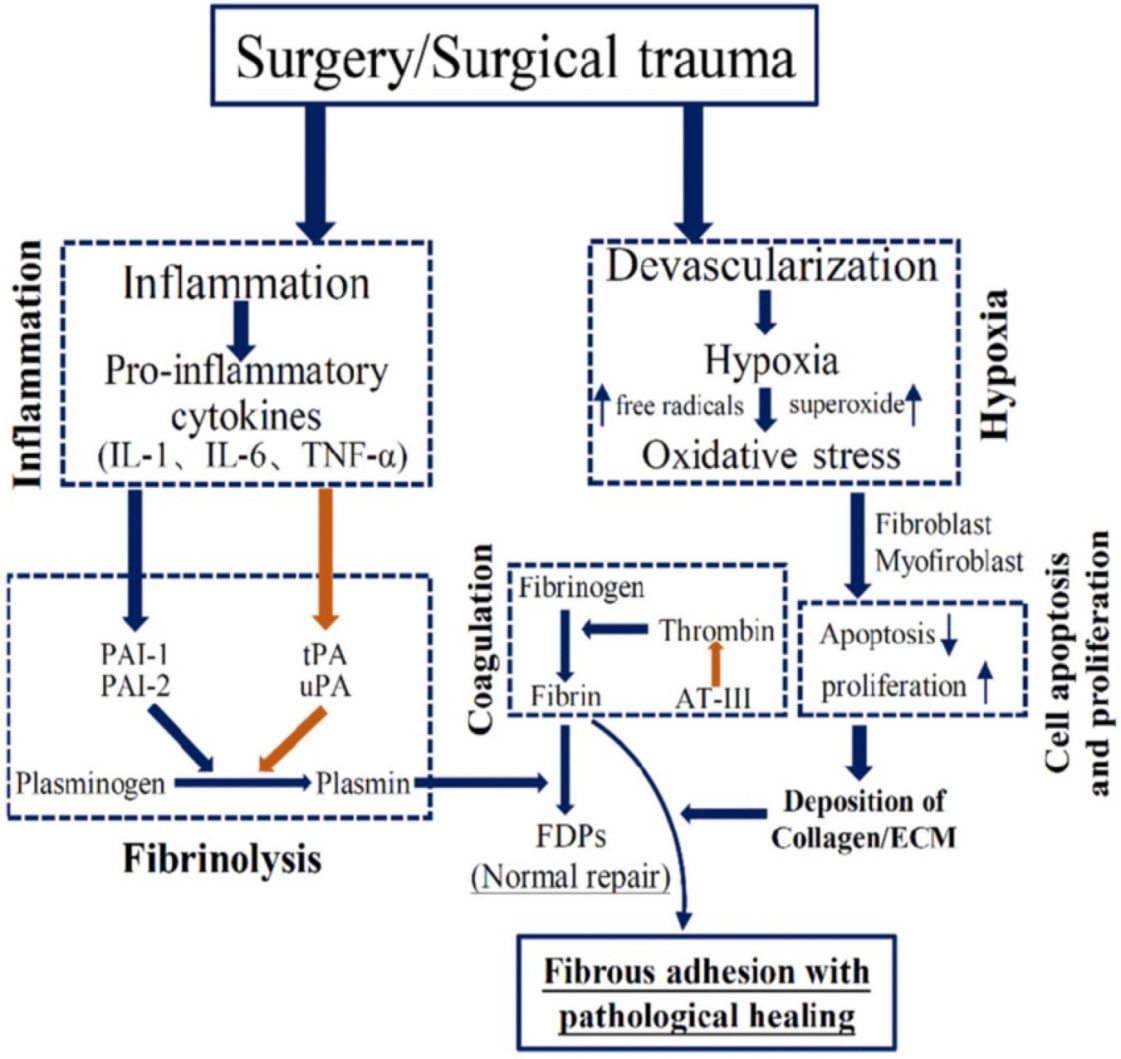
Brief schematic illustration of the pathogenesis of adhesion formation. Reproduced from Hu et al (2021) (15) licensed under CC BY 4.0.

Surgical injury causes the release of cytokines, growth factors, cell adhesion molecules, and histamine, creating an inflammatory response, which can lead to adhesion formation in the peritoneal cavity (1). Adhesions are abnormal tissue attachments, with abdominal surgery being the primary cause, forming within hours post-operation and responsible for 20% of surgical emergencies (2). Figure 1 illustrates the pathogenesis of adhesion formation following surgical trauma. Between 68% and 100% of individuals who undergo laparotomies develop adhesions (3). Adhesive bowel obstruction is a severe complication of abdominopelvic surgeries, especially those involving extensive intra-abdominal manipulation, such as bowel resections, appendectomies, and gynecological procedures. ASBO increases morbidity and mortality risks and can lead to bowel necrosis, with mortality rates as high as 15% during hospitalization (4). Additionally, post-surgical adhesions contribute significantly to increased hospitalization, longer operative times, and extended stays, resulting in a substantial financial burden. Despite available antiadhesive barriers, there is a critical gap in comparing their effectiveness. Seprafilm, a commonly used barrier, has shown limited success, while the novel 4DryField PH gel demonstrates promising results. A direct comparison of these barriers is essential to understand their relative efficacy and risks, as evaluating them against a control (e.g., no barrier or saline) does not offer a complete view of their performance. This study aims to fill this gap by systematically comparing 4DryField PH with Seprafilm, offering valuable insights into their risks and benefits in clinical practice. Although the understanding of adhesion formation mechanisms has improved in recent years, the full pathogenesis and etiology remain unclear, complicating the development of ideal prevention strategies (5).

### Etiology of adhesions

Most individuals with bowel adhesions are asymptomatic. Approximately 75% of symptomatic patients have a history of prior abdominal surgery, while the remaining 25% have experienced intra-abdominal or pelvic inflammatory processes, including malignancies. Symptomatic patients often present with intestinal obstruction (complete or partial), chronic pain, and infertility in females (6).

The use of barrier materials in abdominal surgery is based on their biophysical properties, biocompatibility, proven efficacy, and the goal of preventing adhesion formation to improve patient outcomes and reduce postoperative complications.

### 4DryField PH

It is a powder based on purified potato starch that transforms into a gel with saline solution. The gel then acts as a temporary physical barrier between the surgically traumatized peritoneal surfaces until mesothelial healing is completed and it is subsequently resorbed (7). A gel made of either 5 g 4DF powder and approximately 60 mL saline solution, 10 g 4DF powder, and approximately 120 mL saline solution or 15 g 4DF powder, and approximately 180 mL saline solution, depending on the extent of the wound areas. The gel is evenly distributed on all affected surfaces of the intestine and in the peritoneal cavity (4).

### Seprafilm

a sterile, bioresorbable, temporary translucent adhesion barrier composed of chemically derived sodium hyaluronate and carboxymethylcellulose. It is designed to be absorbed from the peritoneal cavity within seven days and completely excreted from the body within 28 days (8). No adverse or toxic effects have been described with the use of these substances (9).

Promising barrier materials for preventing postoperative adhesions are characterized by several key features. These include the ability to adhere effectively to traumatized and oozing tissues, ensuring proper placement and function. Safety is a critical consideration, ensuring that the material does not cause adverse reactions or complications. The material should also be applicable in laparoscopic procedures, offering versatility in various surgical settings. Additionally, ease of application is important, allowing for efficient and straightforward use during surgery. Postoperative pain management is another consideration, as the material should minimize discomfort for patients after surgery. Finally, cost-effectiveness plays a crucial role, making the material a viable option for widespread clinical use while remaining within budget constraints (10).

### Hypothesis

This review hypothesizes that 4DryField PH is more effective than Seprafilm in preventing adhesions, reducing the incidence and severity of complications such as adhesive small bowel obstruction and chronic pain after abdominopelvic surgery.

### Objective

The objective of this systematic review is to compare the effectiveness of 4DryField PH and Seprafilm in reducing adhesion-related complications, focusing on adhesion incidence, severity, extent, ASBO, and chronic pain from 6 weeks to several years post-surgery.

The primary aims of this review were to evaluate adhesion incidence by measuring the presence of adhesions following surgery and to compare the incidence of adhesive small bowel obstruction (11), the most severe adhesion-related complication. The secondary aims were to assess adhesion severity based on tissue density and the difficulty of surgical dissection, to evaluate adhesion extent by examining the spread of adhesions across different organs or anatomical areas, and to measure the effectiveness of each anti-adhesive barrier in reducing long-term chronic pain.

## Materials and methods

### Study design

This systematic review combined statistical data and qualitative insights to assess outcomes related to adhesion prevention. The methodology adhered to the **Cochrane Handbook** and **PRISMA guidelines**, ensuring transparency, rigor, and reproducibility. The inclusion and exclusion criteria are summarised in Table 1.

**Table 1:** Inclusion and exclusion criteria.

Criteria	Inclusion	Exclusion
Population	Patients aged >18, Male and Female, Abdominopelvic surgery (gynaecological, gastroeneterology, colorectal, urological, non-terminal malignancies and conditions.	Paediatric population and studies with only >65 years, Patients with medical history of inflammatory bowel disease, intrauterine adhesions, and pre-existing terminal conditions.
Intervention	4DryField PH gel	All other Antiadhesive barriers except Seprafilm
Comparator	Seprafilm	All other Antiadhesive barriers except 4DF
Outcomes	Adhesion Incidence, ASBO, Adhesion extent and severity, Chronic Pain >12months	Outcomes unrelated to adhesion-related complications (e.g., non-adhesive small bowel obstruction).
Study	RCT, Prospective and Retrospective Cohort, Observational Studies	Individual/ Isolated Case Studies

### Search methods for study identification

A comprehensive search was conducted between November 2023 and February 2024 across the following databases and registries: **PubMed, Scopus, Cochrane Library, Embase**, and trial registries.

#### Search strategy for seprafilm

(((“CMC” OR “CMC/HA” OR “Seprafilm” OR “Carboxymethylcellulose”) AND (laparo* OR surgery)) AND (pelvic OR abdomin* OR peritoneal)) AND (adhesi*) Filters: Clinical Trial, Comparative Study, Controlled Clinical Trial, Randomized Controlled Trial, from 2019 to 2024 Sort by: Most Recent.

#### Search strategy for 4DryField PH

Since this is a novel anti-adhesive agent, all available results were screened without additional keyword constraints.

### Study selection

A systematic search across multiple databases and clinical registries was conducted, resulting in 437 records (425 from databases and 12 from clinical registries). After removing 172 duplicate records and excluding 113 records that were inaccessible for full-text review, 152 unique records remained. These records were distributed as follows: 38 from PubMed, 10 from Scopus, 24 from the Cochrane Library, 66 from Embase, and 14 from clinical registries. During the screening process, 72 records were excluded based on predefined criteria, including studies involving animal models (*n* = 8), irrelevant studies (*n* = 8), studies with insufficient results (*n* = 34), and studies focused exclusively on malignant conditions (*n* = 11).

After a thorough review of the remaining records, 29 studies were identified that met the inclusion criteria and were considered for further evaluation. These studies were assessed for key factors, including adhesion incidence, the severity and extent of adhesions, chronic pain, and adhesion-related bowel obstruction. Full-text articles for these studies were obtained through individual searches, and reference lists from relevant systematic reviews and meta-analyses were also screened to identify additional studies.

To focus the review on the most relevant data, the search was limited to studies published between 2019 and 2024, reflecting the recent clinical adoption of 4DryField PH, which first appeared in observational studies in 2016.

**Figure 2:**
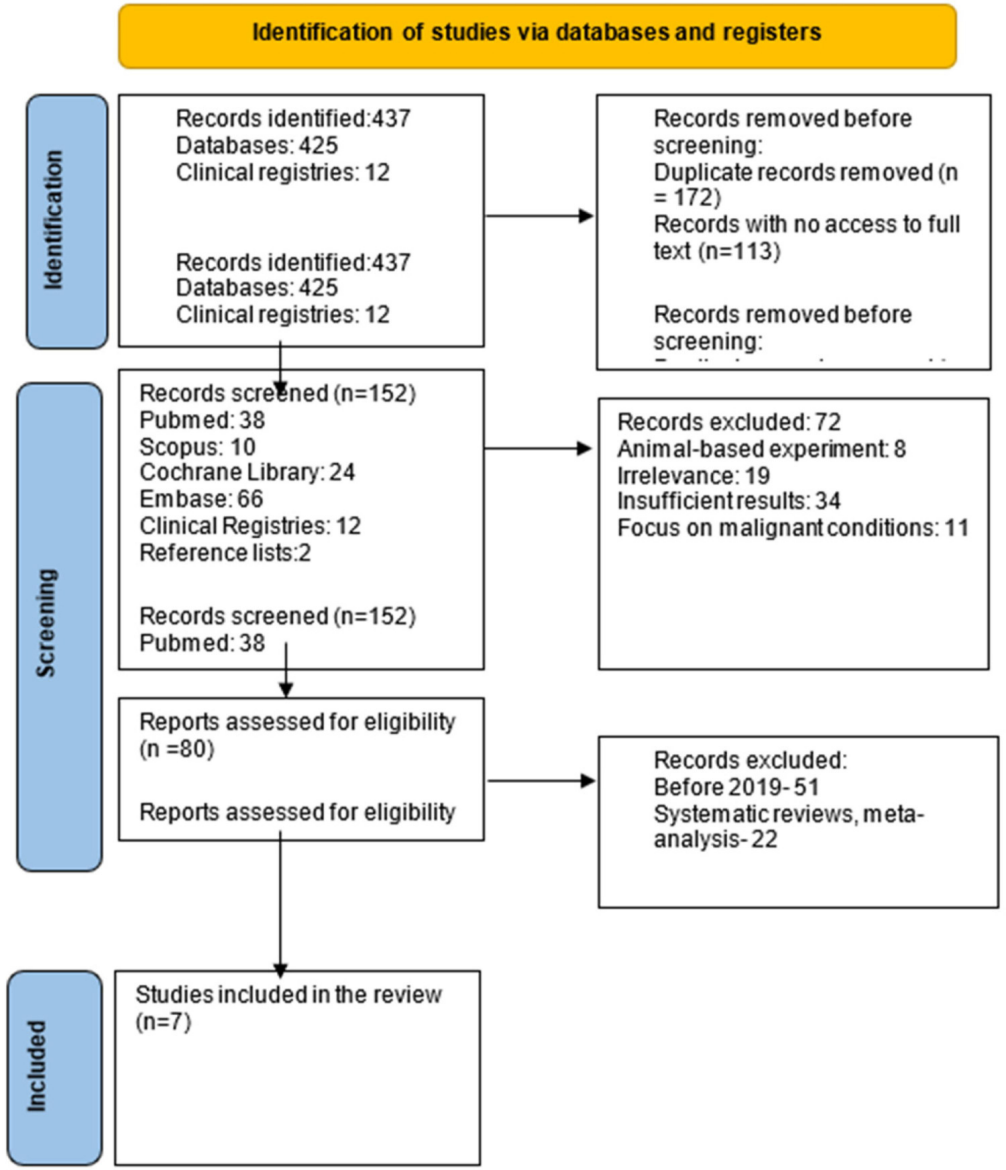
PRISMA flow diagram.

Ultimately, 7 studies met the inclusion criteria and were included in the final analysis. These studies consisted of two randomized controlled trials (RCTs), three retrospective cohort study, and one prospective observational study. Figure 2 shows Preferred reporting items for systematic reviews and meta-analyses (PRISMA) flow diagram.

### Interventions evaluated

The included studies evaluated two anti-adhesive agents: Seprafilm and 4DryField PH. Seprafilm was assessed in three studies and was applied as a sheet. 4DryField PH, on the other hand, was evaluated in four studies and was prepared as a gel by combining powder with a saline solution. The dosage and application techniques for 4DryField PH varied based on the size of the wound area.

### Data extraction and synthesis

Data extraction from the selected studies included essential information such as study ID, year, methods, participants, interventions, outcomes, covariates, and follow-up duration. The data was then synthesized into a narrative summary that highlighted the qualitative findings, focusing on adhesion incidence and the incidence of adhesive small bowel obstruction. In addition, comparative analyses were conducted to evaluate the efficacy of 4DryField PH and Seprafilm, taking into account the types of surgeries, demographics, and follow-up periods. A quantitative meta analysis was considered but ultimately deemed inappropriate because of substantial heterogeneity among the included studies. Surgical indications, study designs, adhesion scoring systems, outcome definitions, and follow up intervals varied widely, and some outcomes were based on second look laparoscopy while others were based on clinical small bowel obstruction. In addition, no study directly compared 4DryField PH with Seprafilm in the same population and several studies lacked comparable control arms. Pooling effect estimates under these conditions would produce misleading summary measures, so a structured narrative synthesis was performed instead.

#### Software used

Data management for this analysis was performed using **RevMan** (Review Manager), a software designed for conducting systematic reviews and meta-analyses. **Excel** was employed for organizing and calculating the primary and secondary outcome data. Statistical analysis, including the calculation of the **Chi-square test**, was initially performed using **AI** tools to facilitate faster computations. These results were subsequently verified and re-calculated using a **manual method** on an online statistical platform to ensure accuracy. **Robvis** was used to generate a visual summary of the **quality assessments** for the included studies, providing a clear overview of the risk of bias. **EndNote** software was used to manage references. Additionally, **ChatGPT** was utilized for editing and improving the readability of the manuscript by removing repeating statements and streamlining the content for clarity.

### Quality assessment

**Figure 3:**
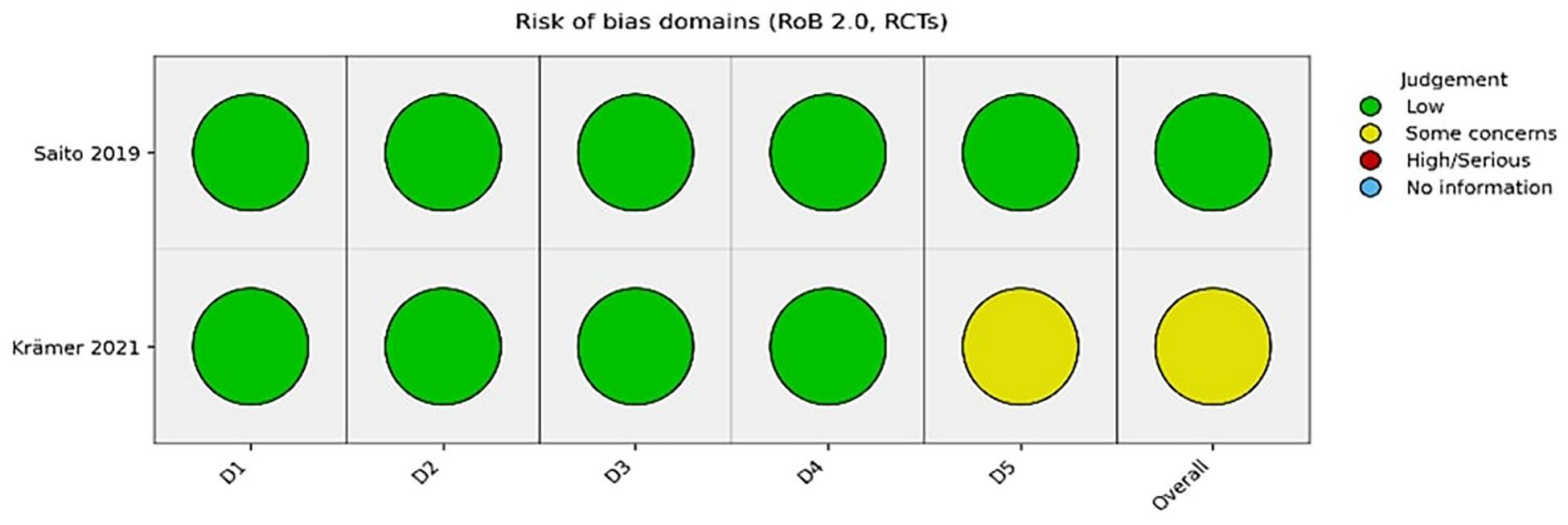
Risk of bias for the included RCTs.

**Figure 4:**
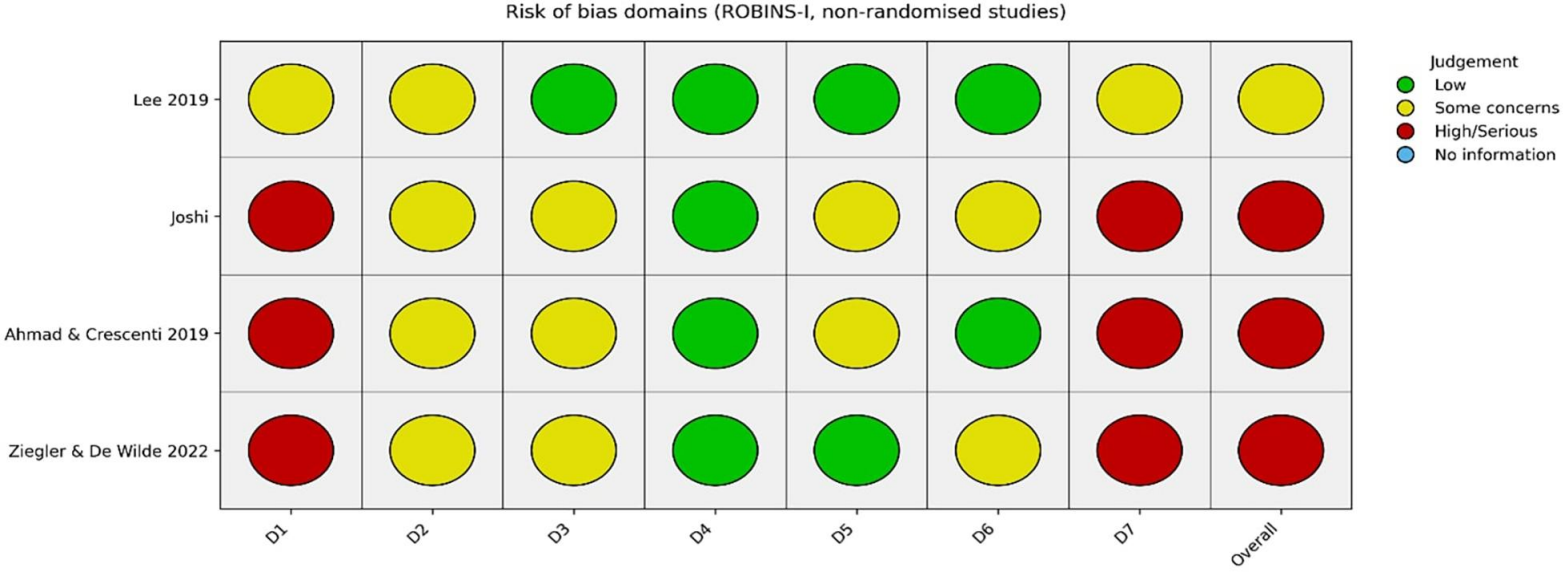
Risk of bias assessment for the non-randomised studies.

The two RCTs demonstrated low to moderate risk of bias overall. Krämer et al. (7) showed low risk across most domains, with “some concerns” relating to selective reporting. Saito et al. (12) was judged to have low risk of bias across all domains. In contrast, most non-randomised studies demonstrated **moderate to serious risk of bias**, primarily due to confounding, lack of randomisation, and selective reporting. Figure 3 summarises the risk of bias for the included RCTs. Figure 4 illustrates the risk-of-bias assessment for the non-randomised studies.

### Ethical considerations

This review utilized publicly available data from published studies, eliminating the need for ethical approval.

## Results

### Outcomes with 4DryField PH

Three studies evaluated the effect of 4DryField PH on adhesion related outcomes.

In the retrospective gynaecological adhesiolysis series by Ziegler and De Wilde, median adhesion severity and extent in the 4DryField PH group decreased from 2 to 0.5 between the first and second operation, representing a substantial reduction in adhesion burden. In contrast, the control group showed an increase in median severity from 1 to 1.5, while median extent remained unchanged at 1. At second look laparoscopy, both severity and extent were lower in the 4DryField PH group than in controls, despite worse baseline adhesions in the 4DryField PH group at the index procedure.

In the randomised controlled trial by Krämer and colleagues in women undergoing endometriosis surgery, 4DryField PH significantly reduced total adhesion scores at second look laparoscopy compared with saline irrigation. The mean total adhesion score at second look was 2.2 in the 4DryField PH group compared with 14.2 in the control group, and the mean number of adhesion affected sites was approximately halved in the 4DryField PH group.

In the cohort reported by Ahmad and Crescenti, 40 patients underwent surgery for adhesive small bowel obstruction with intraoperative application of 4DryField PH. At the index operation, median adhesion severity and extent were III and II respectively, and most patients had dense adhesions between small bowel loops and the abdominal wall. In the eight patients who required reoperation for various reasons, adhesion severity and extent at reoperation were 0 in all but one case. During clinical follow up, no patient developed recurrent adhesive small bowel obstruction.

Taken together, these studies suggest that 4DryField PH is associated with substantial reductions in adhesion severity, extent and overall adhesion burden, particularly in high risk pelvic and ASBO surgery settings.

### Outcomes with seprafilm

Three studies evaluated Seprafilm in colorectal or Hartmann procedures.

In the Hartmann procedure study by Joshi and colleagues, application of Seprafilm at the index operation was associated with a reduction in the degree of adhesions observed at reversal surgery compared with controls. The accessible text describes a decrease in adhesion development in the Seprafilm group, although detailed numeric adhesion scores were not reported.

In the randomised controlled trial by Saito et al. in elective colon cancer surgery, Seprafilm did not significantly reduce the incidence of postoperative small bowel obstruction compared with no barrier. Overall small bowel obstruction occurred in 7.8 percent of patients in the Seprafilm group and 10.6 percent of patients in the control group, and reoperation rates for obstruction were similar in both groups.

In contrast, the retrospective colorectal cohort reported by Lee and colleagues found a lower incidence of postoperative small bowel obstruction in patients treated with Seprafilm compared with those who received no anti adhesion barrier. Small bowel obstruction occurred in approximately 7 percent of patients in the Seprafilm group and 11 percent in the control group. All obstruction episodes in this study were managed conservatively and no patient required surgical intervention. The reduction in small bowel obstruction with Seprafilm was most evident in patients undergoing laparoscopic procedures.

Overall, these findings indicate that Seprafilm may provide a modest reduction in the incidence of postoperative small bowel obstruction in some colorectal surgery settings, although results are not consistent across all studies, and data on second look adhesion scores are limited.

### Three group descriptive comparison

Because the available studies evaluated 4DryField PH and Seprafilm in different populations and with different outcome measures, direct statistical comparison between the two agents was not possible. However, for descriptive purposes, the outcomes of 4DryField PH, Seprafilm and no barrier were considered in a three group framework wherever similar endpoints were reported. A descriptive overview of outcomes across the three groups is presented in Table 2.

**Table 2:** Descriptive overview of outcomes.

Study	Barrier	Adhesion incidence reported	ASBO/SBO reported	Notes
Ahmad & Crescenti 2019 (4)	4DF	Yes (severity/extent ↓ to 0)	Yes (0% recurrent ASBO)	Single-arm ASBO cohort
Ziegler & De Wilde 2022 (13)	4DF	Yes (∼75% reduction)	No	Controlled SLL study
Krämer et al. 2021 (7)	4DF	Yes (85% reduction)	No	RCT with SLL
Krämer et al. 2023 (14)	4DF	No	No	Pain outcomes only
Lee et al. 2019 (9)	Seprafilm	No	Yes (∼7% vs. 11%)	Colorectal cohort
Saito et al. 2019 (12)	Seprafilm	No	Yes (7.8% vs. 10.6%)	RCT
Joshi et al. 2022 (3)	Seprafilm	Yes (qualitative reduction)	No	Hartmann reversal

In studies that used second look laparoscopy to assess adhesions, 4DryField PH consistently produced the lowest adhesion scores at follow up. Seprafilm was not evaluated in these second look laparoscopy studies.

In colorectal surgery studies that used clinical small bowel obstruction as the endpoint, Seprafilm was associated with a lower incidence of obstruction than no barrier in one large cohort, while another randomised trial did not show a significant difference between groups. 4DryField PH was not evaluated in these colorectal small bowel obstruction populations.

In the adhesive small bowel obstruction cohort treated with 4DryField PH, no recurrent ASBO events were reported during follow up, but there was no contemporaneous control group and Seprafilm was not used in this setting.

Viewed together, these patterns suggest that 4DryField PH may be particularly effective at reducing adhesion burden as assessed by second look laparoscopy, whereas Seprafilm has more extensive but modest evidence for reduction of clinically apparent small bowel obstruction. Because these observations are derived from heterogeneous and non overlapping study populations, they are hypothesis generating and should not be interpreted as demonstrating a definitive hierarchy of efficacy between the two agents.

### Exploratory statistical analysis

Exploratory chi square analyses were performed using crude event counts for selected binary outcomes, in order to illustrate the direction of differences between barrier treated groups and control groups.

These analyses, summarised in Table 3, were exploratory and were not intended to constitute a formal meta-analysis, as they did not adjust for differences in study design, patient populations, follow-up duration, or outcome definitions. Instead, they were used to support the broader aim of this systematic review, which was to explore and contextualise comparative patterns between 4DryField PH, Seprafilm, and no barrier in the absence of direct head-to-head studies.

**Table 3:** Exploratory direction of effect.

Outcome	Studies included	Barrier(s)	Comparator	Crude finding	Interpretation
SBO/ASBO	Lee et al. 2019 (9); Saito et al. 2019 (12); Ahmad & Crescenti 2019 (4)	Seprafilm; 4DF	No barrier	Variation across studies	Not comparable due to heterogeneity
Adhesion incidence	Ziegler & De Wilde 2022 (13); Krämer et al. 2021 (7); Joshi et al. 2022 (3)	4DF; Seprafilm	No barrier	Lower scores with 4DF in SLL	Different populations; supportive only

### Secondary outcomes

Secondary outcomes included adhesion severity, adhesion extent, composite adhesion scores and chronic pain. These outcomes were mainly reported in studies of 4DryField PH, as Seprafilm studies rarely included second look scoring or long term pain assessment. Secondary findings from second-look laparoscopy are summarised in Table 4.

**Table 4:** Second look laparoscopy outcomes.

Study	Group	Time to SLL (weeks)	Outcome	First-look score	Second-look score	Summary
Ahmad & Crescenti 2019 (4)	4DF	Variable	Severity/extent	III/II	0/0	Resolved in majority
Ziegler & De Wilde 2022 (13)	4DF	1–56	Severity	2	0.5	Improved
Ziegler & De Wilde 2022 (13)	Control	2–103	Severity	1	1.5	Worsened
Ziegler & De Wilde 2022 (13)	4DF	1–56	Extent	2	0.5	Improved
Ziegler & De Wilde 2022 (13)	Control	2–103	Extent	1	1	No improvement
Krämer et al. 2021 (7)	4DF	5–16	Total adhesion score	—	2.2	Lower
Krämer et al. 2021 (7)	Control	2.9–15.6	Total adhesion score	—	14.2	Higher

In the endometriosis randomised trial by Krämer et al, the total adhesion score and number of affected sites at second look laparoscopy were markedly lower in the 4DryField PH group than in controls, indicating a substantial reduction in both severity and extent of adhesions. In the study by Ahmad and Crescenti, patients treated with 4DryField PH showed a reduction of adhesion severity and extent to 0 at reoperation in nearly all cases, consistent with a near complete resolution of adhesions at the sites evaluated. In the gynaecological adhesiolysis series by Ziegler and De Wilde, 4DryField PH was associated with significant improvements in both severity and extent scores at second look, whereas the control group showed worsening or no change. These secondary outcomes are summarised in Table 4.

Chronic pain was assessed in a follow up study of patients undergoing endometriosis surgery with or without 4DryField PH. Pain scores at one year tended to be lower in the 4DryField PH group than in the control group, suggesting a possible long term benefit for pain, although the difference did not reach conventional levels of statistical significance. Pain outcomes at 12 months are summarised in Table 5.

**Table 5:** Pain outcomes at 12 months.

Group	Participants (*n*)	Baseline mean pain ± SD	12-month mean pain ± SD	Comment
4DF	23	4.5 ± 3.3	2.0 ± 2.3	Trend toward lower pain
Control	23	4.6 ± 3.5	3.4 ± 3.2	Not statistically significant

## Discussion

Postoperative adhesions remain a major source of morbidity following abdominopelvic surgery, contributing to chronic pain, infertility, and adhesive small bowel obstruction (ASBO), with substantial clinical and economic consequences. Although several anti-adhesion barriers are available, direct comparative evidence between individual agents is limited. This systematic review therefore examined the available literature to explore comparative patterns between 4DryField PH and Seprafilm using indirect evidence.

Across the included studies, 4DryField PH was consistently associated with reductions in adhesion burden in settings where second-look laparoscopy was performed, with improvements observed in adhesion severity, extent, and composite adhesion scores. In the ASBO cohort, no recurrent obstruction was reported during follow-up, suggesting potential benefit in high-risk surgical settings where dense adhesions or repeat operations are anticipated. Seprafilm demonstrated modest reductions in postoperative small bowel obstruction in one large colorectal cohort, although this finding was not replicated in a randomised colon cancer trial, and data on second-look adhesion scoring for Seprafilm were limited.

A descriptive three-group framework comparing 4DryField PH, Seprafilm, and no barrier identified consistent patterns rather than a definitive ranking of efficacy. 4DryField PH produced the lowest adhesion scores in second-look laparoscopy studies, while Seprafilm showed variable benefit in colorectal surgery populations when clinical small bowel obstruction was used as the outcome. These findings reflect differences in surgical context, baseline risk, outcome measurement, and follow-up duration across non-overlapping study populations and should be interpreted as comparative signals rather than proof of superiority.

Considerable heterogeneity across studies limited direct comparability and precluded pooled quantitative analysis. Surgical indications, timing and purpose of second-look procedures, adhesion scoring systems, and outcome definitions varied widely, and reporting of barrier preparation and application techniques was inconsistent. While cost-effectiveness analysis was outside the scope of this review, economic considerations such as acquisition cost, operative time, and downstream healthcare utilisation are likely to influence clinical adoption.

Key strengths of this review include its focus on clinically relevant outcomes, inclusion of second-look laparoscopy data, and structured comparison of 4DryField PH, Seprafilm, and no barrier. Exploratory analyses using crude event counts were performed to illustrate the direction of observed differences but were not intended to infer comparative efficacy. The principal limitations remain the small number of studies, methodological heterogeneity, limited long-term follow-up, and the absence of direct comparative trials.

## Conclusion

This systematic review synthesises the available evidence on 4DryField PH and Seprafilm in abdominopelvic surgery in the absence of direct head-to-head trials. The findings suggest that 4DryField PH is associated with consistent reductions in adhesion burden in second-look laparoscopy settings, while Seprafilm may offer modest benefit in reducing postoperative small bowel obstruction in selected colorectal surgery populations.

## Data Availability

The original contributions presented in the study are included in the article/Supplementary Material, further inquiries can be directed to the corresponding author.
